# piRNA: Molecular Mechanisms from Germline Silencing to Somatic Regulation and Roles in Disease

**DOI:** 10.3390/ijms27062685

**Published:** 2026-03-15

**Authors:** Chunmei Zhang, Kexin Yang, Zelong Zhao, Minmin Feng, Linxia Song, Zhenbiao Xu

**Affiliations:** School of Life Sciences and Medicine, Shandong University of Technology, Zibo 255049, China; 18632842808@163.com (C.Z.);

**Keywords:** piRNA, PIWI proteins, transposon silencing, somatic regulation, epigenetics, disease biomarkers

## Abstract

PIWI-interacting RNAs (piRNAs) are a class of small non-coding RNAs initially identified in germline cells as genome guardians that silence transposable elements. Recent studies have expanded this view, revealing that piRNAs and PIWI proteins are broadly expressed in somatic tissues and participate in epigenetic and post-transcriptional gene regulation. This review systematically summarizes piRNA biogenesis and molecular mechanisms, with a focus on their functional diversification from germline to somatic cells. We detail piRNA dysregulation and its association with various human diseases, including cancer, cardiovascular disorders, neurodegenerative diseases, immune dysfunction, and reproductive disorders. By integrating recent findings, this review provides a comprehensive overview of piRNA-mediated regulatory networks and highlights their potential as novel biomarkers and therapeutic targets.

## 1. Introduction

PIWI-interacting RNAs (piRNAs) constitute one of the most abundant classes of small non-coding RNAs in animal cells, typically ranging from 24 to 30 nucleotides in length. Initially identified in 2006 within the germ cells of mice and Drosophila, piRNAs have been classically characterized as guardians of genome integrity through their association with PIWI subfamily Argonaute proteins. These piRNA-PIWI complexes form silencing machineries that repress transposable elements (TEs) via transcriptional gene silencing (TGS) and post-transcriptional gene silencing (PTGS) pathways, thereby maintaining genomic stability and ensuring fertility [1,2,3]. The piRNA pathway has been extensively studied in model organisms, including Drosophila and mice. It is indispensable for gametogenesis, and defects cause infertility and germ cell loss [4,5,6].

The biogenesis of piRNAs involves the processing of long single-stranded precursor transcripts derived from specific genomic loci termed piRNA clusters, which are often enriched in transposon fragments. These precursors undergo complex maturation processes, including phased cleavage and ping-pong amplification cycles, to generate mature piRNAs that guide PIWI proteins to their targets [2,7,8]. Structural studies have identified unique features of PIWI proteins, including non-canonical catalytic tetrads and specialized RNA-binding domains. These features distinguish their target recognition and silencing mechanisms from those of other Argonaute proteins [1,9,10,11]. Notably, the piRNA pathway is tightly regulated at multiple levels, including transcriptional control of piRNA clusters and PIWI proteins, as well as post-translational modifications such as SUMOylation, which facilitate recruitment of heterochromatin machinery to transposon loci [12,13,14,15].

While the germline functions of piRNAs have been well established, emerging evidence over the past decade has expanded their biological significance beyond reproductive tissues. Initially thought to be germline-specific, piRNAs and PIWI proteins are now known to exist in diverse somatic cell types across species, such as neurons, cardiac cells, immune cells, and stem cells [16,17,18,19]. In somatic tissues, piRNAs participate in diverse physiological processes such as cell differentiation, tissue repair, immune regulation, and memory formation. For instance, piRNAs have been implicated in regulating neuronal gene expression during differentiation and synaptic plasticity, as well as modulating immune responses and inflammation [20,21,22]. The presence of piRNAs in somatic stem cells suggests a role in maintaining stemness and guiding differentiation, with PIWI proteins influencing mRNA stability and translation in a piRNA-dependent or -independent manner [18,23]. Moreover, piRNAs have been identified in extracellular vesicles derived from various human body fluids, highlighting their potential as intercellular signaling molecules and non-invasive biomarkers for disease states [24,25]. These findings have prompted a paradigm shift in understanding piRNA biology, recognizing their broader regulatory roles in somatic cells and systemic physiology.

The dysregulation of piRNAs and PIWI proteins has been increasingly associated with a wide spectrum of human diseases. Aberrant piRNA expression profiles have been documented in numerous cancers, including gastric, colorectal, breast, lung, and urological tumors, where they influence tumor initiation, progression, metastasis, and chemoresistance [16,26,27,28,29]. In cancer, piRNAs can act as oncogenes or tumor suppressors by epigenetic and post-transcriptional regulation, targeting pathways including DNA methylation, histone modification, and RNA interference [30,31,32]. Beyond oncology, piRNAs have been implicated in cardiovascular diseases, neurodegenerative disorders, autoimmune diseases, and male infertility, reflecting their involvement in diverse pathological processes [33,34,35,36]. For example, piRNAs promote neuroinflammation and neuronal dysfunction in Alzheimer’s disease, and regulate cardiac differentiation, repair, and vascular remodeling in cardiovascular diseases [14,33,34]. In reproductive medicine, mutations in piRNA biogenesis genes have been linked to spermatogenic failure and male infertility, underscoring the essential role of piRNAs in human germline maintenance [5,37]. Owing to their disease-specific expression profiles and stable presence in biofluids, piRNAs have emerged as dual-function molecules with significant value in both non-invasive diagnostics and targeted therapeutics [24,38,39].

Collectively, the expanding landscape of piRNA research reveals a complex molecular network wherein piRNAs and PIWI proteins orchestrate genome defense, gene regulation, and cellular homeostasis across germline and somatic contexts. The transition from a germline-centric view to recognizing piRNA functions in somatic tissues and disease states has opened new avenues for understanding molecular pathogenesis and developing RNA-based clinical applications. This review aims to comprehensively delineate the molecular mechanisms underlying piRNA biogenesis and function, trace the evolutionary and functional expansion of piRNA pathways from reproductive to somatic cells, and systematically summarize current knowledge on piRNA involvement in human diseases. Furthermore, we discuss the translational potential of piRNAs as biomarkers and therapeutic agents, providing a forward-looking perspective on their clinical utility.

## 2. Biogenesis and Gene Silencing Mechanisms of piRNAs

### 2.1. piRNA Biogenesis Pathways

The biogenesis of piRNAs is a multifaceted and sequential process executed primarily through two interconnected pathways: primary processing and the ping-pong amplification cycle. Primary processing begins with the transcription of long, single-stranded precursors from discrete genomic loci termed piRNA clusters. These precursors are cleaved endonucleolytically by the mitochondrial outer membrane-anchored nuclease Zucchini (Zuc; MitoPLD in mammals), which defines the 5′ ends of primary piRNAs. This cleavage exhibits a sequence preference for a 5′ terminal uridine and is facilitated by accessory factors such as Armitage and Tudor domain-containing proteins, which recruit and stabilize the processing machinery. The resulting pre-piRNAs are trimmed at their 3′ ends by exonucleases such as PNLDC1, then 2′-O-methylated by methyltransferases including HENMT1. These steps produce mature piRNAs that load onto PIWI proteins to form piRNA-induced silencing complexes (piRISCs) [16,40,41].

The ping-pong amplification cycle is a defining feature of piRNA biogenesis in germ cells, particularly in the male germline, serving to amplify piRNAs directed against active transposable elements. In this cycle, piRNA-bound PIWI proteins (e.g., Aubergine (Aub) in Drosophila or MILI in mice) guide the cleavage of complementary transposon transcripts. Cleavage fragments are processed into new piRNA 5′ ends and loaded onto partner PIWI proteins (e.g., AGO3 in flies or MIWI2 in mice). This reciprocal cleavage and loading creates a self-reinforcing loop that amplifies secondary piRNAs. It generates a characteristic 10-nucleotide overlap between sense and antisense piRNAs, enabling efficient silencing of TEs at both transcriptional and post-transcriptional levels [8,40,42]. Tudor domain-containing proteins, such as Tudor, Spindle-E, and TDRD proteins, are critical regulatory factors in this cycle, as they facilitate the assembly of PIWI-piRNA complexes and promote the maturation of pre-piRNAs by recognizing methylated residues on PIWI proteins. This cycle not only amplifies piRNA levels but also enhances the specificity of piRNA-mediated silencing, ensuring effective repression of transposons and other target sequences.

piRNA biogenesis is spatially organized within specialized cytoplasmic granules, including the intermitochondrial cement (IMC), nuage, and processing bodies (P-bodies), where processing factors and PIWI proteins co-localize. For instance, the mitochondrial-anchored protein ASZ1 recruits PIWI proteins to IMC granules, ensuring efficient processing of pachytene piRNAs during spermatogenesis [41,43]. In addition, RNA-binding proteins such as ADAD2 and RNF17 form granules in spermatocytes that are critical for cluster-derived piRNA production, emphasizing the essential role of RNA–protein interactions in piRNA maturation [44].

In somatic cells, piRNA biogenesis pathways are often more variable and simplified compared to the germline. Some somatic piRNAs may be generated independently of the full ping-pong cycle, relying instead on primary processing or alternative routes. For example, piRNA production in Drosophila follicle cells depends largely on primary processing without ping-pong amplification, reflecting tissue-specific adaptations [32,45]. Precursor sources and processing factors may also differ; evidence suggests certain piRNAs originate from mRNA 3′ untranslated regions (3′UTRs) or endogenous coding sequences, with processing potentially coupled to translation dynamics [46,47]. This diversity highlights the evolutionary flexibility of piRNA production, tailored to the distinct requirements of germline and somatic cells.

**Figure 1:**
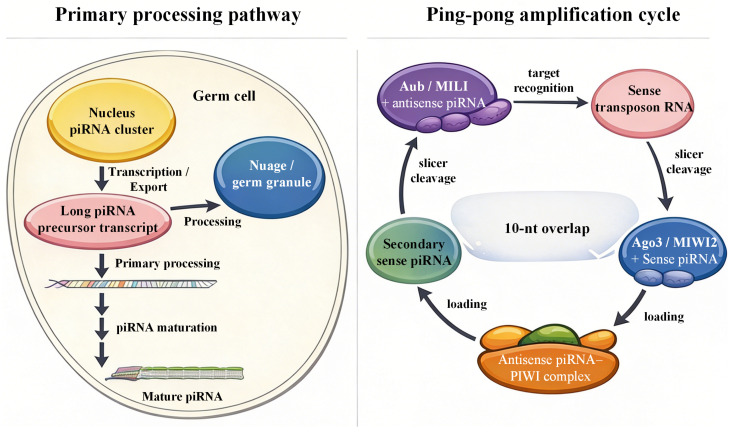
Primary piRNA biogenesis and the ping–pong amplification cycle in germ cells.

In summary, piRNA biogenesis pathways integrate enzymatic cleavage, RNA modification, and protein interactions within specialized subcellular compartments to generate a diverse and abundant piRNA pool. These pathways are tightly regulated in space and time, ensuring robust transposon silencing and genome integrity in the germline while adapting to somatic regulatory demands [8,16,41,42]. This case is depicted in Figure 1.

piRNA clusters in the nucleus produce long precursor transcripts that are exported to cytoplasmic germ granules for processing. The nuclease Zucchini (Zuc; mitochondrial phospholipase D, MitoPLD in mammals) defines the 5′ends of precursor piRNAs (pre-piRNAs), followed by 3′trimming by PNLDC1 (poly(A)-specific ribonuclease-like domain-containing protein 1) and 2′-O-methylation by HENMT1 (HEN1 methyltransferase homolog 1) to generate mature piRNAs. During the ping–pong amplification cycle, antisense piRNA-bound Aubergine (Aub) or MILI (mouse PIWI-like protein 2, PIWIL2) cleave transposon RNAs to produce sense piRNAs that associate with Argonaute 3 (Ago3) or MIWI2 (mouse PIWI-like protein 4, PIWIL4). This reciprocal cleavage generates a characteristic 10-nt overlap between sense and antisense piRNAs, reinforcing transposon silencing and maintaining germline genome stability. Note: Yellow compartment, nucleus containing piRNA clusters or transposon-rich loci; pink oval, long piRNA precursor transcript; blue compartment, nuage (cytoplasmic processing site); purple complex, Aub/MILI bound to antisense piRNA; red oval, sense transposon RNA target; blue complex, Ago3/MIWI2 bound to sense piRNA; orange complex, antisense piRNA–PIWI complex; green oval, secondary sense piRNA. Black arrows indicate transcription, nuclear export, processing, target recognition, piRNA loading, or slicer-mediated cleavage.

### 2.2. Mechanisms of piRNA-Mediated Gene Silencing

piRNAs enforce gene silencing via transcriptional and post-transcriptional mechanisms, primarily targeting transposable elements to preserve genomic stability. In the nucleus, piRNA/PIWI complexes are recruited to complementary genomic loci, where they direct the establishment of repressive chromatin. This recruitment promotes histone modifications such as H3K9me3 (a heterochromatin marker) and may induce DNA methylation at transposon-rich regions under specific conditions [48]. These epigenetic changes lead to compacted chromatin that suppresses transcription, achieving long-term silencing. This transcriptional silencing mechanism is not only critical for maintaining genome stability in germline cells but also contributes to the regulation of transposon activity in somatic tissues, preventing transposon-induced genomic damage and ensuring cellular homeostasis. The specificity of this process is mediated by sequence complementarity between piRNAs and transposon loci, as well as the recruitment of tissue-specific epigenetic modifier complexes [16,32,45].

Post-transcriptionally, piRNA/PIWI complexes operate in the cytoplasm to recognize and cleave transposon-derived RNAs via sequence complementarity. This cleavage, central to the ping-pong cycle, degrades transposon mRNAs, preventing their translation and mobilization. Thus, the ping-pong cycle serves a dual role: amplifying the piRNA pool and enhancing degradation of active transposon transcripts. This provides a dynamic, adaptive defense against TE activity. Notably, piRNA-mediated post-transcriptional silencing occurs in two distinct modes: direct cleavage of target RNAs (when piRNAs form perfect or near-perfect base-pairing with target transcripts) and translational repression (when base-pairing is partial). For partial base-pairing, piRISCs inhibit translation by blocking ribosome recruitment or promoting target mRNA degradation through interactions with translational repressors and RNA decay machinery, allowing fine-tuned regulation of both transposons and host genes [8,40,42].

Beyond transposon repression, piRNA/PIWI complexes also regulate specific host protein-coding genes. This regulation occurs through translational repression or modulation of mRNA stability. For example, piRNAs derived from coding sequences or 3′UTRs can direct PIWI proteins to cleave target mRNAs or regulate their translation, fine-tuning gene expression during spermatogenesis and other developmental processes [32,46,49]. In somatic cells, piRNAs are implicated in regulating metabolism and stress responses, such as modulating long non-coding RNAs and signaling pathways in osteoarthritis. The activity of piRNAs is highly context-dependent, varying across cell types, developmental stages, and disease states. This specificity is achieved through multiple mechanisms: the expression of distinct PIWI family members in different tissues (e.g., PIWIL1/2/4 in germline cells vs. PIWIL3 in somatic tissues), differential processing of piRNA clusters, and interactions between piRISCs and tissue-specific auxiliary proteins. Additionally, post-translational modifications of PIWI proteins (e.g., arginine methylation, phosphorylation) modulate their binding to piRNAs and target sequences, further refining piRNA activity and ensuring appropriate silencing in diverse cellular contexts [50,51].

The interplay between piRNAs and chromatin modifiers is critical for establishing and maintaining silencing. Proteins like Rhino, Deadlock, and Cutoff in Drosophila form complexes that recognize piRNA clusters and facilitate their transcription and processing, linking biogenesis to chromatin state. In addition, post-translational modifications of PIWI proteins (e.g., arginine methylation) affect their interactions with Tudor domain-containing proteins and the assembly of processing granules, thus regulating silencing efficiency [13,52,53,54].

Beyond their well-characterized role in germline transposon silencing, piRNAs have also been reported to exhibit transposon silencing activity in selected somatic tissues, although the authenticity, scope, and biological significance of this function in mammalian systems remain topics of active investigation and debate. Evidence from recent studies demonstrates that piRNAs are expressed in a variety of somatic tissues, including the brain, heart, liver, and cancer tissues, where they contribute to the repression of transposon activity and the maintenance of somatic genome integrity [36,55].

Notably, somatic piRNA-mediated transposon silencing differs mechanistically from that in germline cells. In somatic tissues, piRNA biogenesis relies more on the primary piRNA pathway rather than the full ping-pong cycle, with distinct PIWI family members (e.g., PIWIL3 in mammals) mediating silencing. Additionally, somatic piRNAs often target a subset of transposons that are active in somatic cells, rather than the broad range of transposons targeted in germline cells. This tissue-specific targeting is driven by the expression of tissue-specific piRNA clusters and the differential recruitment of epigenetic modifiers by piRISCs [17,32,56].

The biological significance of somatic piRNA-mediated transposon silencing is multifaceted: it prevents transposon-induced genomic rearrangements and mutations, regulates the expression of host genes adjacent to transposon loci, and contributes to cellular homeostasis. In disease states such as cancer, dysregulation of somatic piRNA expression can lead to aberrant transposon activation, which promotes genomic instability and tumor progression. Thus, somatic piRNA-mediated transposon silencing is a critical component of piRNA function that complements their germline roles and underscores their broader biological relevance [57,58].

piRNA pathways also exhibit epigenetic inheritance, whereby maternally deposited piRNAs can guide transposon silencing in progeny, ensuring transgenerational genome defense. This inheritance involves the maintenance of heterochromatic marks and piRNA cluster activity, highlighting piRNAs’ role in epigenetic memory [59,60]. At the same time, piRNA pathways have mechanisms that prevent abnormal permanent silencing. These mechanisms protect endogenous genes from uncontrolled RNA interference and show that piRNA systems can both promote and restrict silencing [61].

**Figure 2:**
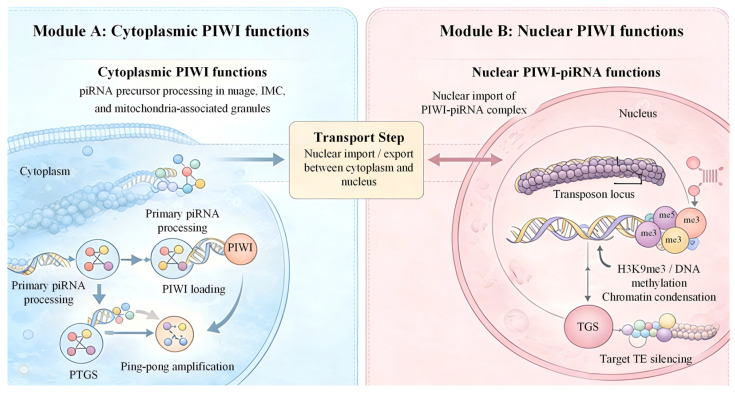
Compartmentalized piRNA biogenesis and PIWI-mediated silencing in the cytoplasm and nucleus.

In conclusion, piRNA-mediated silencing encompasses chromatin remodeling, RNA cleavage, translational control, and epigenetic inheritance. Together, these mechanisms maintain genome integrity, regulate gene expression, and support cellular homeostasis in both germline and somatic tissues [16,32,46,50]. This case is depicted in Figure 2.

This schematic illustrates the spatial organization of the PIWI–piRNA pathway. In the cytoplasm, piRNA precursors are processed within nuage (perinuclear germ granule), intermitochondrial cement (IMC), or mitochondria-associated granules, followed by PIWI loading, primary piRNA maturation, and ping–pong amplification. Cytoplasmic PIWI (P-element-induced wimpy testis protein)–piRNA complexes mediate post-transcriptional gene silencing (PTGS) through cleavage of transposon RNAs and repression of target transcripts. In the nucleus, PIWI–piRNA complexes recognize transposon loci and recruit heterochromatin machinery, promoting H3K9me3 (histone H3 lysine 9 trimethylation) deposition, DNA methylation, and chromatin condensation to establish transcriptional gene silencing (TGS). Note: Blue-shaded region, cytoplasm; pink-shaded region, nucleus; central box, nuclear import/export of PIWI–piRNA complexes between cytoplasm and nucleus; arrows indicate the direction of molecular processing, transport, or regulatory action.

## 3. Functions of piRNAs in the Male Reproductive System and Testicular Health

### 3.1. Maintaining Germline Genome Integrity

Safeguarding genome integrity in the germline is essential for the accurate transmission of genetic information across generations. The PIWI-interacting RNA pathway constitutes a primary defense mechanism against the mobilization of transposable elements such as LINE1 and intracisternal A-particle (IAP) elements during spermatogenesis. Unchecked TE activity can cause insertional mutations and genomic instability, compromising gamete quality and offspring viability. piRNAs associate with PIWI proteins to form effector complexes that silence active transposons via both transcriptional and post-transcriptional mechanisms. Genetic ablation of key piRNA pathway components (e.g., MIWI and MILI) in mice causes severe transposon de-repression, meiotic arrest, impaired spermatogenesis, and total male infertility. This directly demonstrates the pathway’s essential role in fertility [62]. Mechanistically, piRNA-guided PIWI complexes cleave complementary transposon transcripts and induce repressive epigenetic marks such as H3K9me3 at transposon loci, thus inhibiting their transcription [63,64]. piRNA precursors are derived from genomic clusters enriched with transposon fragments and are subject to complex epigenetic regulation involving chromatin remodelers to ensure germline-specific expression [65,66]. In humans, mutations in PIWIL genes or abnormal piRNA expression are associated with male infertility, including non-obstructive azoospermia and severe oligozoospermia. This highlights the clinical importance of the piRNA pathway in testicular function [62]. Moreover, the piRNA pathway extends beyond transposon silencing to regulate endogenous gene expression programs critical for spermatogenic progression [67]. Collectively, these findings establish the piRNA pathway as a core molecular mechanism that preserves germline genome integrity, thereby safeguarding fertility and genomic stability across generations.

### 3.2. piRNAs as Biomarkers of Testicular Function and Disease

Beyond their canonical role in germline transposon silencing, piRNAs have emerged as promising biomarkers for assessing testicular function and diagnosing male infertility. piRNAs are not limited to intracellular compartments. They are stably present in extracellular biofluids such as seminal plasma and blood, and their expression correlates with key sperm parameters, including concentration and motility [68]. Specific piRNAs, including piR-823 and piR-015520, exhibit altered abundance in the semen or serum of infertile men, suggesting their utility as non-invasive or minimally invasive indicators of spermatogenic status [39]. These molecules may aid in distinguishing obstructive from non-obstructive azoospermia, conditions with distinct etiologies and clinical management. piRNAs are stable in biofluids due to their binding with PIWI proteins and 2′-O-methylation at the 3′ end, making them highly suitable as reliable biomarkers [58]. Their tissue-specific expression patterns further support diagnostic specificity by minimizing confounding signals from other tissues. Moreover, piRNAs regulate gene expression and epigenetic states in germ cells. They can thus serve as markers not only for spermatogenic output but also for molecular defects underlying infertility [62]. Current research leverages high-throughput sequencing and sensitive detection platforms to establish piRNA signatures reflective of testicular health [69]. Although the field continues to evolve, integrating piRNA-based assays into reproductive medicine holds promise for improving diagnosis, prognosis, and personalized management of male infertility.

## 4. Expression and Regulatory Functions of piRNA in Somatic Cells

### 4.1. Discovery and Characteristics of Somatic piRNAs

High-throughput sequencing has substantially expanded our understanding of piRNA biology, revealing their presence beyond the germline into diverse somatic tissues, including brain, heart, immune organs, and epithelia. Initially, piRNAs were considered germline-specific small RNAs that function in transposon silencing. However, growing evidence shows that somatic piRNAs are widespread, though they are generally less abundant than those in the germline [33,70]. Somatic piRNAs exhibit distinct genomic origins and sequence features compared to germline piRNAs. Germline piRNAs mainly come from canonical piRNA clusters enriched in transposable element sequences. In contrast, somatic piRNAs often originate from alternative genomic regions, such as intergenic loci and the antisense strands of protein-coding genes [70,71]. Their sequence characteristics—including length distribution and nucleotide biases—diverge from germline piRNAs, reflecting potential differences in biogenesis pathways and functional specialization. The expression of PIWI proteins such as HIWI (*PIWIL1*) in human somatic cells supports the existence of functional piRNA pathways outside the germline [70]. Mechanistically, somatic piRNA biogenesis relies more on primary processing than on the ping-pong amplification cycle (which is dominant in germ cells). This is supported by the lack of typical ping-pong signatures in somatic piRNA populations [72]. Additionally, there are somatic-specific regulatory mechanisms. For example, in Drosophila follicle cells, the transcription factor Traffic jam regulates piRNA pathway components and the flamenco piRNA cluster [72,73]. Somatic piRNAs have also been detected in extracellular vesicles and exosomes, suggesting potential roles in intercellular communication [21]. Collectively, these findings establish somatic piRNAs as a distinct class of regulatory small RNAs with specialized biogenesis and functional attributes tailored to somatic cellular environments.

### 4.2. Regulatory Functions of Somatic piRNAs

**Figure 3:**
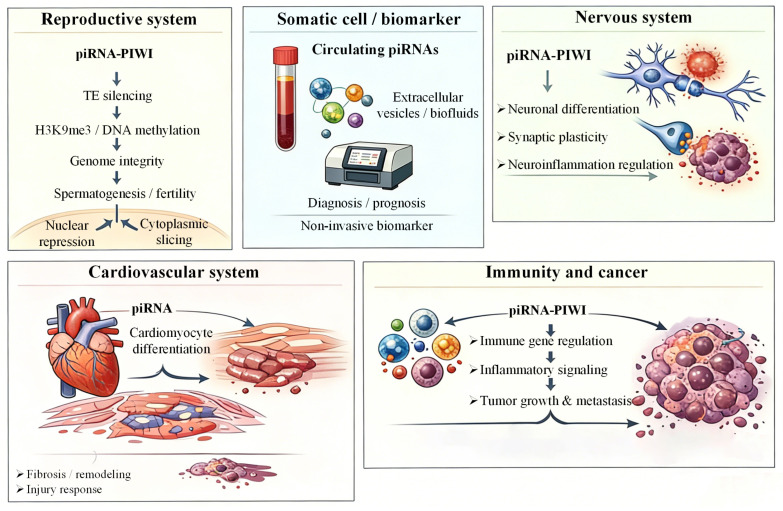
Functional roles of piRNAs in the reproductive system and diverse somatic tissues.

Beyond transposon repression, somatic piRNAs function as versatile regulators of gene expression, targeting specific host mRNAs and modulating diverse biological processes across multiple tissues. In the nervous system, piRNAs have been reported to regulate neuronal differentiation and synaptic plasticity. For example, they interact with cold-shock domain-containing RNA-binding proteins to affect the expression of neuronal markers such as MAP2 and TUBB3. This suggests their involvement in neurodevelopment and potential roles in neurodegenerative disease mechanisms [20,70]. In cardiovascular tissues, piRNAs contribute to epigenetic regulation of cardiac progenitor cell differentiation and cardiomyocyte gene expression programs, with dysregulation correlating with cardiovascular disease pathology [33]. Somatic piRNAs also take part in cellular stress responses and metabolic regulation. Under oxidative stress or DNA damage, specific piRNAs show altered expression patterns, which may regulate DNA repair pathways and cellular homeostasis [23,74]. Additionally, piRNAs have been implicated in immune regulation, with aberrant expression associated with autoimmune and infectious conditions, suggesting roles in modulating immune responses through epigenetic and post-transcriptional mechanisms [21]. In cancer biology, piRNAs and PIWI proteins are increasingly recognized for their involvement in tumorigenesis, cancer stem cell maintenance, and chemoresistance [75,76], with dysregulated piRNA expression profiles serving as potential diagnostic and prognostic biomarkers [26,36,77]. Functional studies in model organisms further show that somatic piRNAs regulate developmental processes, such as sexual differentiation, growth, and programmed cell death. This highlights their broad physiological significance [78,79,80]. Collectively, these insights reveal that somatic piRNAs constitute a multifaceted regulatory network influencing gene expression, epigenetic landscapes, and cellular responses to environmental cues, thereby contributing to tissue homeostasis and disease pathogenesis. This case is depicted in Figure 3.

Although piRNA expression has been detected in various somatic tissues, the genuine biological origin and function of such somatic piRNAs remain highly controversial in the field. A key scientific question is as follows: do the piRNAs identified in somatic cells by high-throughput sequencing represent authentic functional transcripts? Or do they merely come from germline leakage, random degradation fragments produced during apoptosis, or other small RNAs misannotated by bioinformatic methods (e.g., tRNA-derived fragments) [36,81]? Overall, although increasing evidence suggests regulatory roles of somatic piRNAs, the functional validation in mammalian systems remains limited, and many reported piRNAs require further biochemical confirmation.

Current studies show obvious contradictions. On the one hand, several studies using rigorous biochemical experiments have confirmed that some somatic piRNAs can bind PIWI proteins and exert regulatory functions [17,82,83]. On the other hand, piRNA levels in most mammalian somatic cells are significantly lower than those in germ cells. Furthermore, somatic cells generally lack essential PIWI protein combinations (e.g., Ago3 and Aub) and key cofactors for the ping-pong cycle [16,18]. This leads to the absence of canonical secondary piRNA amplification and the dominance of primary biogenesis pathways.

Therefore, future studies urgently need to establish stricter criteria for somatic piRNA identification. For instance, a combination of 2′-O-methylation detection, PIWI protein immunoprecipitation, and loss-of-function models can be used to distinguish authentic functional piRNAs from non-specific background fragments [84,85].

The piRNA–PIWI pathway, composed of PIWI proteins and piRNAs, regulates gene expression in both germline and somatic systems. In the reproductive system, piRNA–PIWI complexes silence transposable elements through RNA cleavage and epigenetic repression, promoting H3K9me3 (histone H3 lysine 9 trimethylation) deposition, DNA methylation, and genome stability during spermatogenesis. In somatic contexts, piRNAs participate in diverse biological processes, including neuronal differentiation, synaptic plasticity, and neuroinflammation in the nervous system, cardiomyocyte differentiation and cardiac remodeling in the cardiovascular system, and immune regulation and tumor progression in cancer-related processes. In addition, circulating piRNAs detected in extracellular vesicles or biofluids may serve as non-invasive biomarkers for disease diagnosis and prognosis.

## 5. The Role of piRNA in Nervous System Development and Disease

### 5.1. Functions of piRNA in Neurodevelopment and Plasticity

PIWI-interacting RNAs (piRNAs) and their associated PIWI proteins were first identified in the germline for transposon silencing. They have since emerged as critical regulators in the mammalian brain, especially during neurodevelopment and synaptic plasticity. In the mammalian brain, piRNAs and PIWI proteins exhibit dynamic expression patterns throughout neurogenesis, neuronal migration, and synaptogenesis, implicating them in neural circuit formation [86]. *Piwil2* (Mili) is essential for maintaining the fitness and proper differentiation of adult neural progenitor cells in the postnatal hippocampus. Depletion of *Piwil2* impairs neuronal differentiation, induces cellular senescence, and promotes reactive gliosis. This highlights its role in sustaining neurogenesis and brain plasticity during aging [86]. In human embryonal carcinoma NT2 cells undergoing neuronal differentiation, specific piRNAs such as DQ582359 and DQ596268 are upregulated and interact with cold-shock domain-containing RNA-binding proteins (DIS3, DIS3L2, YB-1) to modulate neuronal markers MAP2 and TUBB3, with overexpression enhancing differentiation [20]. PIWIL4 expression increases during retinoic acid-mediated neuronal differentiation. Knockdown of *PIWIL4* suppresses neuronal marker expression by blocking the removal of repressive H3K27me3 marks from neuronal gene promoters. This indicates a key role in chromatin remodeling during neurogenesis [87]. Beyond neurons, piRNA pathways contribute to maintaining neuroglial homeostasis, as Mili depletion in the hippocampus generates reactive glia [86,88]. Collectively, these findings demonstrate that piRNAs and PIWI proteins orchestrate neurodevelopmental processes through epigenetic, transcriptional, and post-transcriptional mechanisms.

### 5.2. piRNA Dysregulation in Neurodegenerative and Psychiatric Disorders

Aberrant piRNA expression has been increasingly implicated in the pathogenesis of neurodegenerative and psychiatric disorders, highlighting their potential as biomarkers and therapeutic targets. In Alzheimer’s disease (AD), significant piRNA alterations occur in affected regions such as the hippocampus and prefrontal cortex, with dysregulated piRNAs potentially targeting pathways involving amyloid precursor protein (APP) and tau protein [89]. Parkinson’s disease (PD) and amyotrophic lateral sclerosis (ALS) show disease-specific piRNA profiles. In PD, these profiles correlate with α-synuclein aggregation; in ALS, they relate to mitochondrial function genes. This suggests piRNA-mediated regulation affects protein aggregation and energy metabolism, which are central to neurodegeneration [89]. In psychiatric illnesses such as schizophrenia and depression, altered piRNA expression may affect neurotransmitter systems and stress response pathways through epigenetic modifications and transposon activation. Loss of piRNA-mediated transposon silencing can lead to genomic instability and neuroinflammation, processes that exacerbate neuronal dysfunction [90]. piRNAs have also been detected in glial cells and may modulate the neuroimmune environment, further influencing disease progression [91]. Mechanistically, piRNAs may contribute to disease by regulating gene expression through epigenetic modifications, RNA stability, and interaction with RNA-binding proteins, thereby affecting neuronal survival, synaptic function, and neuroinflammation [92,93]. Emerging evidence identifies piRNAs as molecular links between neurodevelopmental regulation and neurodegenerative pathology. Dysregulation disrupts neuronal homeostasis and triggers pathological cascades that lead to cognitive decline and psychiatric symptoms [91]. Consequently, piRNA pathways represent promising avenues for developing diagnostic biomarkers and RNA-based therapeutic strategies.

## 6. The Role of piRNA in the Cardiovascular System and Other Somatic Diseases

### 6.1. piRNA in Cardiovascular Homeostasis and Disease Regulation

PIWI-interacting RNAs, originally identified for their role in germline transposon silencing, have emerged as critical regulators in cardiovascular biology, influencing heart homeostasis and disease pathogenesis. In cardiac tissue, piRNAs regulate fundamental cellular processes, including cardiomyocyte differentiation, hypertrophy, apoptosis, and autophagy. These processes underlie both developmental programming and pathological remodeling [94,95]. For instance, piR-210512 is upregulated in myocardial infarction (MI) models, suggesting involvement in modulating cardiomyocyte survival post-injury, potentially through interactions with epigenetic modifiers or signaling pathways governing cell fate decisions [94].

Aberrant piRNA expression profiles have been linked to various cardiovascular diseases (CVDs), including heart failure, ischemia–reperfusion (I/R) injury, pulmonary arterial hypertension (PAH), and aortic valve calcification. These piRNAs may function by modulating key signaling pathways, including TGF-β and NF-κB. These pathways are central to inflammation, fibrosis, and hypertrophic responses in the myocardium [96]. In addition, piRNAs regulate cardiac fibroblast activation and extracellular matrix remodeling. These processes are critical for cardiac fibrosis and heart failure progression, often through epigenetic mechanisms such as DNA methylation and histone modification [97,98].

Circulating piRNAs have garnered attention as promising biomarkers for early diagnosis and prognosis of CVDs due to their remarkable stability in body fluids, often encapsulated within extracellular vesicles [96]. Their differential expression in plasma and serum correlates with disease severity and prognosis in MI and heart failure, allowing non-invasive detection and monitoring [99,100]. Moreover, piRNAs represent potential therapeutic targets; modulating their expression could attenuate maladaptive cardiac remodeling. For example, targeting heart necroptosis-associated piRNA (HNEAP) has been shown to reduce cardiomyocyte death and preserve cardiac function following ischemic injury [101]. Collectively, piRNAs are integral to maintaining cardiovascular homeostasis and modulating disease progression through regulation of cardiomyocyte fate, fibroblast activity, and inflammatory signaling, positioning them as valuable biomarkers and therapeutic targets in cardiovascular medicine.

### 6.2. piRNA Functions in Immune Diseases and Cancer

piRNAs extend their regulatory influence beyond the cardiovascular system, playing significant roles in immune diseases and cancer. Within the immune system, piRNAs are expressed in various immune cells, including T lymphocytes and macrophages, where they contribute to fine-tuning immune responses. Dysregulation of piRNA expression has been implicated in autoimmune diseases such as systemic lupus erythematosus (SLE) and rheumatoid arthritis (RA), as well as in immunodeficiency and infectious conditions [102]. Mechanistically, piRNAs may regulate inflammatory cytokine and immune checkpoint molecule expression via transcriptional and post-transcriptional control. This modulates immune cell activation, tolerance, and inflammation [103].

In oncology, piRNAs exhibit dualistic roles, functioning either as tumor suppressors or oncogenes depending on cellular context and cancer type [76]. As tumor suppressors, piRNAs can silence oncogenes or metastasis-promoting genes, inhibiting tumor growth and dissemination. Conversely, some piRNAs act as oncogenic factors by repressing tumor suppressor genes, promoting cancer cell proliferation, invasion, and chemoresistance [98]. Aberrant piRNA expression has been documented in multiple malignancies including gastric, breast, and colorectal cancer, where expression profiles correlate with tumor aggressiveness and patient prognosis [104]. piRNA/PIWI protein complexes modulate gene expression through epigenetic modifications, mRNA stability regulation, and translational control, contributing to the complex regulatory networks driving tumor biology [17].

Notably, tumor-derived piRNAs are secreted into the extracellular space via exosomes and other vesicles. They enter the circulation and serve as easily accessible biomarkers for cancer detection and monitoring. These circulating piRNAs hold promise for liquid biopsy applications, enabling early cancer screening, tumor classification, and assessment of therapeutic response with minimal invasiveness [82]. The stability of piRNAs in bodily fluids and their cancer-specific expression profiles enhance their clinical utility as diagnostic and prognostic tools. Collectively, piRNAs represent a critical layer of gene regulation in immune function and oncogenesis, underscoring their potential as novel biomarkers and therapeutic targets in immune-related disorders and cancer management [This case is depicted in Table 1a,b].

## 7. Clinical Translation Potential of piRNAs: Challenges and Prospects

### 7.1. piRNA as Biomarkers for Disease Diagnosis and Prognosis

The tissue-specific expression patterns and disease associations of PIWI-interacting RNAs have positioned them as promising candidates for non-invasive biomarkers across multiple pathologies, including neurodegenerative disorders, cardiovascular diseases (CVD), cancers, and infertility. The presence of piRNAs has been confirmed across multiple biological fluids, including but not limited to circulation, cerebrospinal fluid, seminal fluid, and vesicle-enclosed compartments. This enables the development of fluid-based diagnostic panels for early detection and prognosis [108,109]. In neurodegenerative diseases such as Alzheimer’s and Parkinson’s, piRNAs show differential expression in biofluids. For example, piR-hsa-327831 and piR-hsa-1968818 have high diagnostic sensitivity and specificity for Parkinson’s disease [89,109]. In CVD, altered piRNA expression correlates with myocardial hypertrophy, heart failure, and myocardial infarction, supporting their utility in non-invasive diagnosis and prognosis [111,112,113]. In oncology, piRNAs have significant diagnostic and prognostic potential. Serum piR-020619 and piR-020450 have been validated as accurate early detection biomarkers for colorectal cancer. Meanwhile, piR-823 acts as a prognostic marker in colorectal, renal, and breast cancers [80,105,106,114]. Liquid biopsy approaches leveraging extracellular vesicle-associated piRNAs further enhance non-invasive cancer diagnostics [24,115]. High-throughput sequencing combined with machine learning has allowed the construction of disease-specific piRNA signatures. These signatures improve diagnostic sensitivity and enable subtype classification and risk stratification. Machine learning models using piRNA sequence descriptors have achieved over 90% diagnostic accuracy in breast and colorectal cancers [116,117]. Despite these advances, challenges remain in validating piRNA biomarkers across large clinical cohorts to ensure reproducibility, and standardization of detection methodologies is critical for clinical translation [116]. Overall, integrating piRNA profiling with advanced computational methods holds promise for developing robust non-invasive diagnostic and prognostic tools across diverse diseases.

Although piRNAs hold considerable potential as diagnostic and prognostic biomarkers, a number of methodological hurdles impede their effective translation into clinical practice. To begin with, bias in small RNA sequencing persists as a key problem: the detection of piRNAs can be affected by library construction methods, read lengths, and mapping stringency, which in turn results in inconsistent quantitative outcomes across different research studies [118,119]. Secondly, verifying the subcellular and tissue localization of piRNAs is fraught with difficulties, which poses a challenge in differentiating functionally active piRNAs from degraded fragments or contaminating sequences [120]. Thirdly, the inconsistent annotation of piRNAs across various databases complicates the comparison of research results, as many sequences lack uniform identifiers or a clear genomic source [81]. Lastly, the absence of standardized normalization approaches, such as suitable reference genes and data processing pipelines, undermines the reproducibility of results in large-scale clinical cohorts. Resolving these limitations will enhance the accuracy, reliability, and clinical translatability of piRNA-based biomarkers [121].

### 7.2. Therapeutic Targeting of piRNAs: Strategies and Challenges

Therapeutic strategies targeting piRNAs focus on modulating their function to correct aberrant gene regulation in disease pathogenesis. Approaches include antisense oligonucleotides (ASOs) or small molecule inhibitors to suppress pathogenic piRNAs, and delivery of piRNA mimics or viral vectors to restore protective functions. PIWI proteins are essential effectors of piRNA-mediated silencing. Therefore, targeting them with small molecule inhibitors or degraders is a promising approach, especially in cancers with piRNA pathway dysregulation [111,112,122]. For example, piRNA-823 promotes tumor growth and chemoresistance in multiple myeloma through the TGF-β1-mediated AKT/ERK pathway. This suggests that targeting this piRNA or its associated pathways could improve therapeutic efficacy [107]. Similarly, modulating piRNAs involved in cardiac fibrosis and neurodegenerative diseases offers potential for novel treatments [81,110]. However, substantial obstacles remain for clinical translation, requiring cautious interpretation of therapeutic prospects. First, efficient and tissue-specific delivery remains a major hurdle. Systemic administration often leads to poor accumulation in target tissues, and current delivery systems such as nanoparticles and viral vectors remain limited by low transfection efficiency, immune clearance, and inconsistent biodistribution [123]. Second, off-target effects represent a critical safety concern. piRNA mimics or inhibitors may inadvertently disrupt endogenous RNA regulatory networks, leading to unintended gene silencing or activation and potentially causing cellular dysfunction [124]. Third, germline safety represents a unique and serious constraint for systemic therapies. Given the indispensable roles of piRNAs in maintaining genome stability and fertility in germ cells, non-specific targeting may damage gonadal function, disrupt gametogenesis, or induce heritable genomic alterations, raising substantial ethical and clinical safety concerns [62]. Moreover, the understanding of piRNA regulatory networks in somatic cells remains incomplete, necessitating further research into piRNA biogenesis, target specificity, and interactions with other non-coding RNAs. piRNA-mediated epigenetic and post-transcriptional regulation is complex, requiring precise modulation to avoid unintended consequences. Furthermore, the heterogeneity of piRNA expression across diseases and individuals complicates the design of universal therapies. Advances in delivery technologies, such as nanoparticle-based systems and exosome-mediated transport, alongside improved molecular characterization of piRNA pathways, will be pivotal in overcoming these obstacles. In summary, while piRNAs and PIWI proteins represent attractive therapeutic targets, translating these strategies into safe and effective clinical interventions requires addressing delivery, specificity, and mechanistic knowledge gaps through multidisciplinary research. This case is depicted in Table 1a,b.

## 8. Conclusions

The exploration of piRNAs has evolved from their initial characterization as germline genomic guardians to their recognition as multifaceted regulators in diverse somatic tissues. This expanded understanding establishes piRNAs as pivotal epigenetic and post-transcriptional modulators of both transposable elements and host gene expression, broadening their biological significance while challenging researchers to reconcile mechanistic insights across varying cellular contexts. This case is depicted in Table 2.

piRNAs maintain cellular homeostasis through a complex balance between transposon silencing and gene regulation. While classical studies emphasized germline protection, emerging evidence reveals piRNAs fine-tune somatic gene expression networks influencing development, differentiation, and stress responses—a functional duality requiring context-dependent interpretation [17,102].

Clinically, aberrant piRNA expression links to male infertility, neurodegenerative disorders, cardiovascular diseases, cancers, and immune conditions, positioning them as central players in disease pathophysiology. However, heterogeneity across diseases and patient populations challenges universal biomarker or therapeutic target delineation, necessitating rigorous validation across diverse cohorts.

The exceptional stability of piRNAs in biofluids positions them as revolutionary non-invasive tools for disease diagnosis, prognostic assessment, and therapeutic surveillance. Their extracellular resilience enables longitudinal studies invaluable for disease management, though clinical translation requires overcoming detection sensitivity, specificity, and standardization hurdles through harmonized validation efforts.

Therapeutically, targeting piRNAs or PIWI proteins offers precision medicine prospects, yet remains nascent with obstacles in delivery systems, off-target effects, and safety. Interdisciplinary collaboration integrating nanotechnology, molecular biology, and pharmacology is essential for developing safe and effective piRNA modulators.

Future research must prioritize tissue-specific piRNA biogenesis and molecular mechanisms, particularly how piRNAs interface with disease networks. To tackle these existing limitations and promote the advancement of the field, four specific future research priorities are put forward: First, it is crucial to standardize piRNA annotation procedures, as this will help resolve discrepancies in sequence identification and genomic origins across different databases, thereby enhancing the reproducibility of piRNA-related research and facilitating comparisons between various studies. Second, there is a need to conduct large-scale, multi-center clinical validation trials to confirm the diagnostic, prognostic, and therapeutic potential of piRNA-based biomarkers and therapeutic approaches, which will help surmount the current obstacles in clinical translation. Third, in-depth exploration of the functional mechanisms of piRNAs in somatic tissues is required to further our comprehension of their biogenesis pathways, target specificity, and interactions with other regulatory molecules, so as to clarify their real biological functions and settle the existing disputes in this field. Fourth, single-cell piRNA profiling, by utilizing advanced single-cell sequencing techniques, can assist in mapping the cell-type-specific expression patterns of piRNAs, thus revealing their roles in cellular heterogeneity and disease progression. Comprehensive interaction profiling will identify critical nodes for therapeutic targeting, bridging fundamental research and clinical application through innovative manipulation and detection technologies.

In conclusion, piRNA research stands at a pivotal juncture, with expanding knowledge revealing extensive regulatory capacities and clinical relevance. Synthesizing diverse findings is essential to translate insights into health benefits, with continued interdisciplinary efforts enabling integration of piRNA-based diagnostics and therapeutics into precision medicine, enhancing our capacity to diagnose, monitor, and treat human diseases.

## Figures and Tables

**Table 1:** (a) Dysregulated piRNAs/PIWI proteins in human cancers. (b) Dysregulated piRNAs in non-cancer diseases.

(**a**)						
**Disease Category**	**Disease**	**Dysregulated** **piRNA/PIWI**	**Expression Change**	**Representative** **Mechanism**	**Clinical** **Significance**	**Reference**
Cancer	Colorectal cancer	piR-020619, piR-020450, piR-823	piR-020619, piR-020450 downregulated; piR-823 upregulated	Regulate tumor proliferation and metastasis; associated with epigenetic regulation (DNA methylation and histone modification) and activation of epithelial–mesenchymal transition (EMT) pathways	piR-020619 and piR-020450 may serve as early diagnostic biomarkers; piR-823 may function as a prognostic biomarker and potential therapeutic target	[26,31,105,106]
Cancer	Gastric cancer	*PIWIL1*	Upregulated	Promotes gastric cancer cell proliferation, migration, metastasis, and tumorigenesis in a piRNA-independent manner; associated with global transcriptomic reprogramming	Potential diagnostic and prognostic biomarker for gastric cancer	[30]
Cancer	Ovarian cancer	piR-823	Upregulated	The piR-823/*PIWIL1*/*DNMT3B*/*CDH1* signaling axis promotes epithelial–mesenchymal transition (EMT) and tumor stemness	Potential diagnostic biomarker and therapeutic target	[75]
Cancer	Multiple myeloma	piR-823	Upregulated	Activates the TGF-β1-mediated AKT/ERK signaling pathway, promoting cell proliferation and chemoresistance	Targeting piR-823 may improve chemotherapy sensitivity	[107]
Cancer	Lung adenocarcinoma	Specific piRNA clusters	Aberrantly expressed	Associated with cancer stem cell maintenance and inhibition of apoptosis pathways	piRNA-based models may assist early detection of lung adenocarcinoma	[80]
(**b**)						
**Disease** **Category**	**Disease**	**Dysregulated** **piRNA/PIWI**	**Expression** **Change**	**Representative** **Mechanism**	**Clinical Significance**	**Reference**
Neurodegenerative diseases	Alzheimer’s disease	Hippocampus- and prefrontal cortex-associated piRNAs	Aberrantly expressed	May affect APP and tau-related pathways, promote neuroinflammation, and disrupt transposon silencing and neuronal homeostasis	piRNAs detected in cerebrospinal fluid or blood may serve as potential diagnostic biomarkers	[34,89,91,108]
Neurodegenerative diseases	Parkinson’s disease	piR-hsa-327831, piR-hsa-1968818	Aberrantly expressed	May regulate α-synuclein aggregation and mitochondrial function-related gene expression	Peripheral blood piRNAs may serve as diagnostic biomarkers for Parkinson’s disease	[89,98,109]
Neurodegenerative diseases	Amyotrophic lateral sclerosis (ALS)	Disease-associated piRNA clusters	Aberrantly expressed	May influence mitochondrial function and neuronal RNA metabolism	Potential diagnostic biomarkers and targets for mechanistic studies	[89,91,93]
Cardiovascular diseases	Myocardial infarction (MI)	piR-210512, HNEAP	piR-210512 upregulated; HNEAP aberrantly expressed	piR-210512 regulates cardiomyocyte survival; HNEAP inhibits m5C methylation of Atf7 mRNA and mediates cardiomyocyte necroptosis	Circulating piR-210512 may serve as a diagnostic biomarker for MI; targeting HNEAP may reduce cardiomyocyte death	[94,101]
Cardiovascular diseases	Myocardial fibrosis/heart failure	CFAPIR; fibrosis-associated piRNA clusters	CFAPIR downregulated; fibrosis-associated piRNAs upregulated	CFAPIR suppresses myocardial fibrosis via MBNL2-related regulation; other piRNAs activate TGF-β/NF-κB signaling and fibroblast activation	Potential therapeutic target for myocardial fibrosis; circulating piRNAs may have prognostic value in heart failure	[97,99,100,110]
Cardiovascular diseases	Pulmonary arterial hypertension (PAH)	Vascular-associated piRNA clusters	Aberrantly expressed	Regulate vascular remodeling and endothelial/smooth muscle cell proliferation	Potential diagnostic biomarkers and therapeutic targets	[96,99,100,111,112]
Immune diseases	Systemic lupus erythematosus (SLE)/rheumatoid arthritis (RA)	Immune cell-associated piRNA clusters	Aberrantly expressed	Regulate inflammatory cytokine expression and immune cell activation, contributing to loss of immune tolerance	Peripheral blood piRNAs may reflect disease activity and serve as potential immunomodulatory targets	[21,102]
Immune diseases	Chagas disease (*Trypanosoma cruzi* infection)	piRNAs targeting IL-6 signaling	Downregulated	Reduced suppression of the IL-6 pathway may lead to excessive inflammation and cardiac fibroblast injury	Dysregulated piRNAs may serve as early diagnostic biomarkers for infection	[102,103]
Reproductive diseases	Male infertility (non-obstructive azoospermia)	piR-823, piR-015520; *PIWIL* gene variants	Aberrantly expressed; increased mutation burden in *PIWIL* genes	piRNA pathway defects impair transposon silencing, leading to spermatogenic arrest and dysregulated spermatogenesis genes	piR-823 and piR-015520 in semen or serum may serve as diagnostic biomarkers; PIWIL genes are potential pathogenic targets	[5,37,62]

**Table 2:** Summary Table of piRNA Molecular Mechanisms, Functions, and Disease Associations.

Topic	Key Contents and Mechanisms	Primary Functions	Associated Diseases/Applications
1. piRNA Biogenesis and Molecular Mechanisms	Primary processing and ping-pong cycle; mediated by nucleases such as Zucchini; involves mitochondrial anchor proteins, Tudor domain-containing proteins, etc.	Formation of piRISC complexes; silencing of transposons via TGS and PTGS; maintenance of genomic stability	Germ cell dysfunction, male infertility
2. Functions of piRNA in the Reproductive System	Silencing of transposons such as LINE1 and IAP; involvement of PIWI proteins including MIWI and MILI; regulation of spermatogenesis-associated genes	Maintenance of genomic integrity in germ cells; ensuring proper spermatogenesis and fertility	Non-obstructive azoospermia, oligozoospermia, male infertility
3. Expression and Regulation of piRNAs in Somatic Cells	Derived from intergenic regions, mRNA 3′UTRs, etc., dependent on primary processing; interact with cold-shock domain-containing proteins, DIS3, and others	Regulation of neuronal differentiation, cardiomyocyte fate, immune responses, and stem cell maintenance	Neurodevelopmental disorders, cardiovascular diseases, immune disorders
4. Roles of piRNA in the Nervous System	Regulation of neuronal genes such as MAP2 and TUBB3; involvement in epigenetic remodeling; maintenance of neural progenitor cell function	Modulation of synaptic plasticity, neuronal differentiation, and neuroinflammation	Alzheimer’s disease, Parkinson’s disease, schizophrenia
5. Roles of piRNA in the Cardiovascular System and Other Diseases	Regulation of TGF-β and NF-κB signaling pathways; modulation of cardiomyocyte autophagy, apoptosis, and fibrosis; regulation of immune checkpoint molecules	Modulation of cardiac remodeling, vascular remodeling, and immune tolerance	Myocardial infarction, heart failure, pulmonary arterial hypertension (PAH), rheumatoid arthritis, cancer
6. Clinical Translation Potential	Serve as liquid biopsy biomarkers (serum, seminal plasma, exosomes); utilized in machine learning-assisted diagnostics; therapeutic targeting strategies (ASOs, piRNA mimics)	Early disease diagnosis, prognostic assessment, therapeutic targets	Cancer, neurodegenerative diseases, cardiovascular diseases, male infertility

## Data Availability

Not applicable. This review is a synthesis and analysis of published literature and did not generate or analyze new experimental data.
